# The reproducibility of cardiac T2* measurement in thalassaemia major patients using bright and black blood sequences

**DOI:** 10.1186/1532-429X-11-S1-P283

**Published:** 2009-01-28

**Authors:** Gillian Smith, Taigang He, John Paul Carpenter, Raad Mohiaddin, David Firmin, Dudley Pennell

**Affiliations:** Royal Brompton Hospital, London, UK

**Keywords:** Cardiovascular Magnetic Resonance, Iron Overload, Thalassaemia Major, Iron Loading, Myocardial Iron

## Introduction

Regular blood transfusions are required for the long term survival of patients with thalassaemia major (TM) but this can cause tissue iron overload. Myocardial iron overload can lead to heart failure and subsequent death. Iron chelation therapy can reduce tissue iron levels but efficacy needs to be closely monitored. Ferritin levels and hepatic iron loading do not correlate well with cardiac iron levels thus the need for a reliable, non-invasive means of follow-up. Cardiovascular magnetic resonance (CMR) using a bright blood gradient echo sequence has been validated for the quantification of tissue iron levels. Signal intensity decreases with increasing echo time and the rate of decay is proportional to iron loading. This bright blood sequence has been widely used for clinical follow-up and efficacy of pharmacological intervention. A T2* <20 ms is considered indicative of cardiac iron overload. Contrast between blood pool and myocardium for this technique is however relatively low, and blood signal artifacts which propagate onto the myocardium can further reduce accuracy. A double inversion pulse black blood sequence has recently been developed to address these issues. It is of interest therefore to assess reproducibility in a larger group of patients and to compare differences according to iron overload levels.

## Purpose

To evaluate the comparative reproducibility of a bright blood and recently available black blood T2* sequence for patients with different myocardial iron loading levels.

## Methods

100 consecutive TM patients, 50 with a T2* value <20 ms and 50 patients with a T2* > 20 ms were selected (51 female, age 27 years – range 11 to 50) All patients were scanned using a 1.5 T Siemens Sonata scanner with a 6 channel phased array cardiac coil and ECG gating. A mid ventricular short axis slice was imaged using both the bright blood and black blood sequences. Both sets of data were analysed twice by the same operator. A mono-exponential decay curve was derived using Thalassaemia tools (CMRtools, Cardiovascular Imaging Solutions, London, UK). For patients with high iron loading and longer echo time intensities points falling below the noise level were removed to improve the curve fit (truncation method). Bland-Altman analysis was used to show the intra-operator reproducibility for all patients and subgroups.

## Results

**Figure 1:**
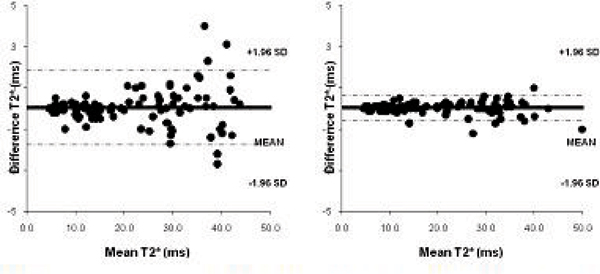
**Intra-operator reproducibility of bright blood** ***(left)*** **and black blood** ***(right)*** **acquisitions for all patients**.

**Figure 2:**
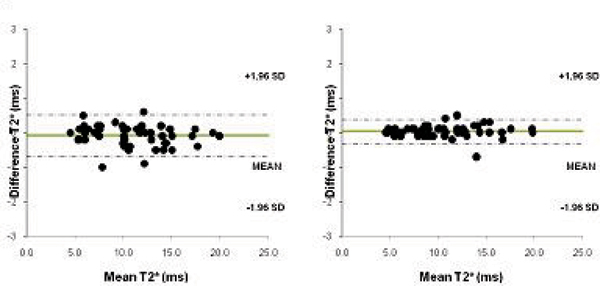
**Intra-operator reproducibility of bright blood** ***(left)*** **and black blood** ***(right)*** acquisitions for patients with T2* values <20 ms.

**Figure 3:**
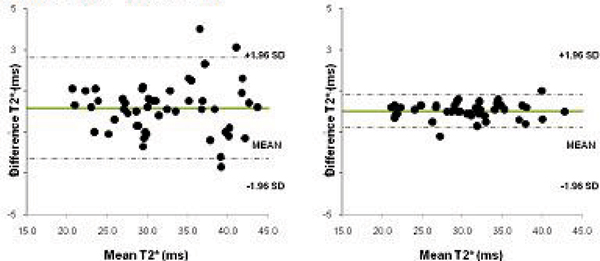
**Intra-operator reproducibility of bright blood** ***(left)*** **and black blood** ***(right)*** acquisitions for patients with T2* values >20 ms.

T2* values ranged between 4.5–43.8 ms (mean 21.6 ± 12 ms) for all patients. For the 50 iron overloaded patients values ranged from 4.5–19.9 ms (mean 10.9 ± 3.8 ms) and for the patients with T2* values over 20 ms the range was 21.1–43.8 (mean 32.3 ± 6.4 ms). Figure 1 shows the Bland-Altman plot of T2* values obtained for the two measurements for the bright blood and dark blood acquisitions. The coefficient of variability was 4.23 for bright blood and 1.48 for dark blood. For patients with a T2* value of <20 ms the coefficient of variability was 2.81 (bright blood) vs 1.68 (dark blood – Figure 2) and for >20 ms 3.88 vs 1.29 (Figure 3).

## Conclusion

This study shows improved reproducibility across all T2* values for the black blood sequence in comparison with the previously validated bright blood sequence.

